# De novo 11q13.3q13.4 deletion in a patient with Fanconi renotubular syndrome and intellectual disability: Case report and review of literature

**DOI:** 10.3389/fped.2023.1097062

**Published:** 2023-04-21

**Authors:** Yingxiao Shen, Xiaoqin Xu, Jiansong Chen, Jingjing Wang, Guanping Dong, Ke Huang, Junfen Fu, Dingwen Wu, Wei Wu

**Affiliations:** ^1^ Department of Endocrinology, The Children’s Hospital, Zhejiang University School of Medicine, National Clinical Research Center for Child Health, Hangzhou, China; ^2^ Department of Orthopedics, The Children’s Hospital, Zhejiang University School of Medicine, National Clinical Research Center for Child Health, Hangzhou, China; ^3^ Department of Nephrology, The Children’s Hospital, Zhejiang University School of Medicine, National Clinical Research Center for Child Health, Hangzhou, China; ^4^ Department of Genetics and Metabolism, Genetics and Metabolism, The Children’s Hospital, Zhejiang University School of Medicine, National Clinical Research Center for Child Health, Hangzhou, China

**Keywords:** deletion 11q13, copy number variation, fanconi renotubular syndrome, SHANK2, ANO1, mental retardation, developmental delay

## Abstract

**Objective:**

To explore the genetic etiology of a child with facial dysmorphia, developmental delay, intellectual disability, Fanconi renotubular syndrome, and Chiari malformations.

**Materials and methods:**

Whole exome sequencing (WES), Copy number variation sequencing (CNV-seq), and mitochondrial gene detection (Long-PCR + NGS) were applied to detect possible pathogenic mutations and chromosomal copy number variations (CNVs), together with databases and literature reviews to clarify the pathological significance of the candidate mutations.

**Results:**

The WES revealed a 2.10 Mb interstitial deletion from 11q13.3 to 11q13.4, which was later confirmed by CNV-seq involving 11 OMIM genes, among which SHANK2, DHCR7, NADSYN1, FADD, NUMA1, IL18BP, ANO1, and FGF3 are disease-causing. The mitochondrial gene shows no variations.

**Conclusion:**

The child has carried a *de novo* 11q13.3q13.4 microdeletion, in which SHANK2 genes may be the key gene responsible for the phenotype of intellectual disability. The renal manifestation of the child, which can be diagnosed as Fanconi renotubular syndrome, has an unknown cause but may result from the effect of the ANO1 gene. This case adds a new phenotype to the deletion of this region.

## 1. Introduction

Copy-number variant (CNV) is a type of structural variant in the human genome, which is defined as a segment of DNA that is 1 kb or larger and is present at a variable copy number in comparison with a reference genome (1). CNVs can be classified into insertions, deletions, and duplications (1). Some of these variants are thought to be responsible for phenotypic abnormalities in humans caused by haploinsufficiency when involving dosage-sensitive genes. CNVs often occur in regions reported as containing or being flanked by large homologous repeats or segmental duplications, which are called rearrangement hotspots (2). There is growing evidence showing the role of large duplications and deletions in causing various specific genetic disorders, which are characterized as microdeletion and microduplication syndromes (e.g., Angelman syndrome, Di George syndrome, Charcot-Marie-Tooth disease, etc.) (3).

Here, we report the identification of a 2.1 Mb deletion in chromosome 11q13.3q13.4 spanning from 69603687 to 71717599 in a child with global developmental delay, intellectual disability, Fanconi renotubular syndrome, hypophosphatemic rickets, and Chiari **I** malformation. Possible genetic causes of these phenotypes are discussed.

## 2. Methods

### 2.1. Subject

The female proband, aged 13 years and 6 months, is the first child of healthy, unrelated parents *via in vitro* fertilization. Her mother has a history of miscarriage because of ectopic pregnancy. She was born prematurely at 36 weeks of gestation by cesarean section due to fetal arrest, with a birth weight of 2.4 kg (<2SD) and a length of 46 cm (<−2SD) and has a history of birth asphyxia (Apgar score was unknown). Since the infant period, she has been noted as shorter than children of her age and sex. At the age of 1, her length was 70 cm (−2SD∼−1SD), and since then, she has grown by 3–5 cm per year; currently, at age 13 (137 cm height, <3SD; 36 kg weight, −2SD∼−1SD), the proband still exhibits global developmental delay. The patient was slow in achieving milestones and had speech delay. Specifically, she took her first step independently at 24 months. Up until now, it has been hard for her to stand on a single foot, and her running posture is not coordinated. She couldn’t use simple words like “papa” and “mama” until 1 1/2 years old. She is able to communicate with her family members, though there is little logic in her conversation, but her social skills are lacking. She underwent a children’s social life ability test (S-M scale), which includes six aspects: self-help, locomotion, occupation, communication, socialization, and self-direction. The results showed that she has only a moderate level of social functioning. Because of this, it was difficult for her to adapt to normal school life and she is currently attending a special education school. She also has severe intellectual disability (IQ score < 40 in the Merriam-Webster intelligence test). The patient’s family history on both the maternal and paternal sides is negative for congenital anomalies or developmental delay, except that her mother’s grandfather died of renal cancer. Her father is 178 cm in height and her mother is 157 cm.

Four years ago, she was misdiagnosed in a local hospital with hypophosphatemic rickets due to decreased blood phosphate levels, increased alkaline phosphatase levels, and short stature and was treated with sodium phosphate. She was then found to have renal tubular acidosis during follow-up care, along with an impaired renal glucose threshold, nephrogenic diabetes insipidus, hypercalciuria, hypophosphatemia, and proteinuria (especially low-molecular-weight proteins, like α 1 microglobulin), which was clinically diagnosed as Fanconi Syndrome by a nephritic physician. Currently, she is being treated with oral medications including calcitriol, phosphorus, and potassium citrate/sodium citrate.

In the past ten years, she has suffered from recurrent perianal and buttock abscesses and has undergone the incision and drainage of abscesses several times.

**Figure 1:**
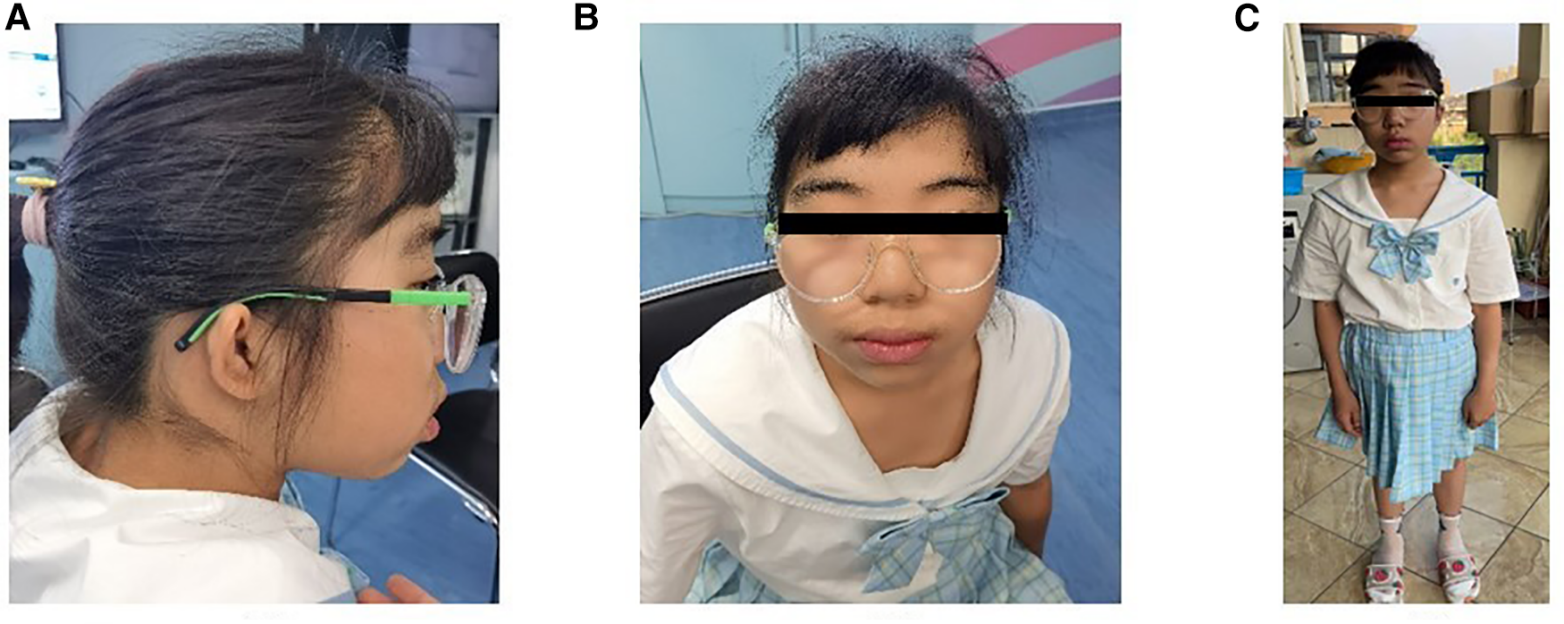
Proband aged 13 years and 6 months. (**A**) profile (**B**) frontal (**C**) full-body.

Physical examination revealed ocular hypertelorism, thick eyebrows, a broad nose, anteverted nares, and a long philtrum (Figure 1) but no noticeable limb deformities. She has normal enamel development, B3-B4 stage of both breasts and PH2 stage of the vulva, though clitoral hypertrophy. Until her last visit, menarche had not yet occurred.

Two recent laboratory results are shown in Table 1. Before that, the patient was tested for 24 h urinary protein quantity (2022-03-10). The results showed a 24 h urine output of 6200 ml, 24 h urinary protein of 3816.1 mg/24 h, and total trace protein of 615.5 mg/l. An immune system examination revealed decreased IgG and C3, but other immunoglobulins and lymphocyte subsets were normal.

**Table 1:** Laboratory.

	2022-4-22	2022-6-7
Blood pH	7.3	7.295
Serum creatinine	63 mmol/L	66 umol//L
Serum calcium	2.22 mmol/L	2.15 mmol/L
Serum phosphate	1.31 mmol/L	1.11 mmol/L
Urine routine	sugar(++++), protein(+−), pH 7.5, specific gravity 1.009	sugar(++++), protein(+), pH 8.0, specific gravity 1.014
PTH	65.8 pg/ml	141.2 pg/ml
Urine creatinine	1100 umol/L	1417 umol/L
25-hydroxyvitamin D	33.8 mmol/L	31.8 mmol/L
Urine calcium/urine creatinine	0.22	0.38
Alkaline phosphatase	825 U/L	837 U/L
Urine calcium	0.68 mmol/L	1.51 mmol/L
Urine phosphate	–	15.67 mmol/L
Urinary phosphorus excretion rate	–	65.7%

**Figure 2:**
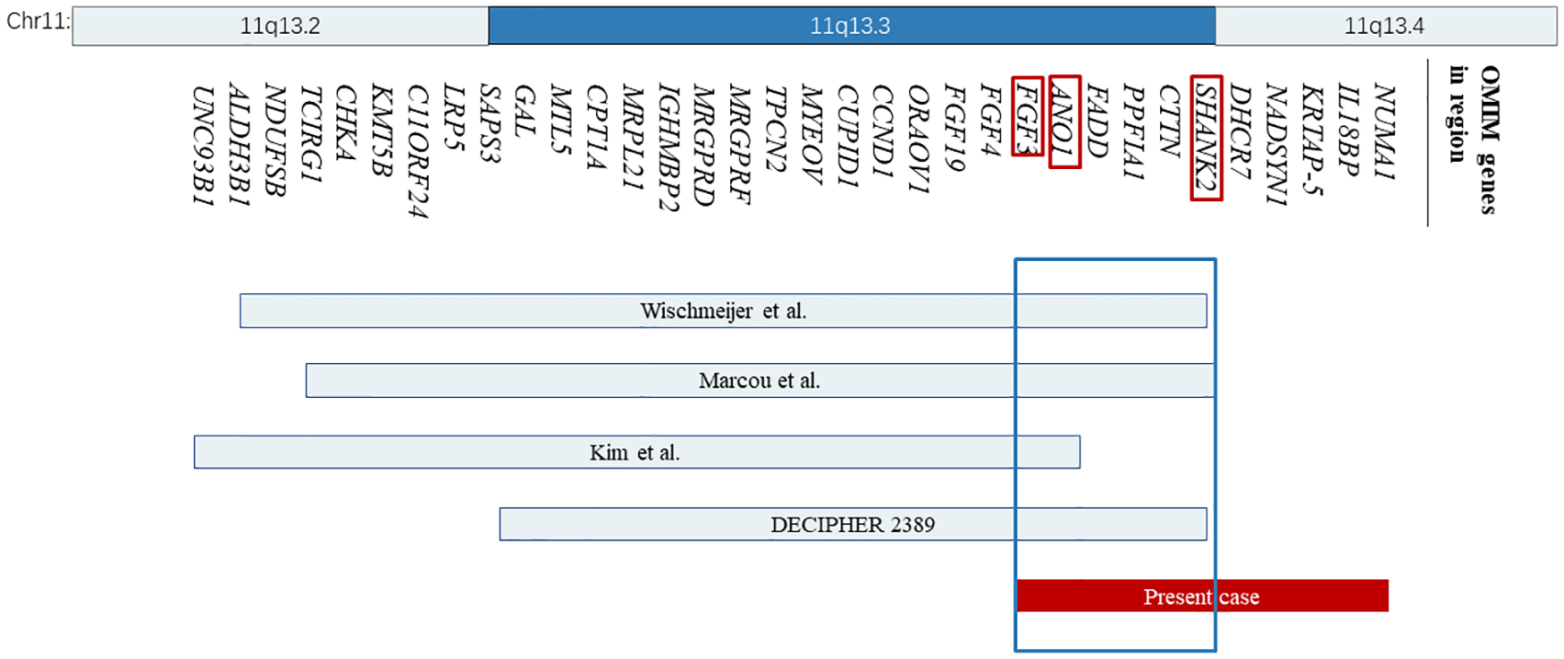
The location and size of the individual chromosome 11q13 deletions are shown for the reported cases with respect to our present case. The 34 OMIM genes deleted in these cases are represented with bands 11q13.2 and 11q13.4 shown on the top. OMIM, Online Mendelian Inheritance in Man.

**Figure 3:**
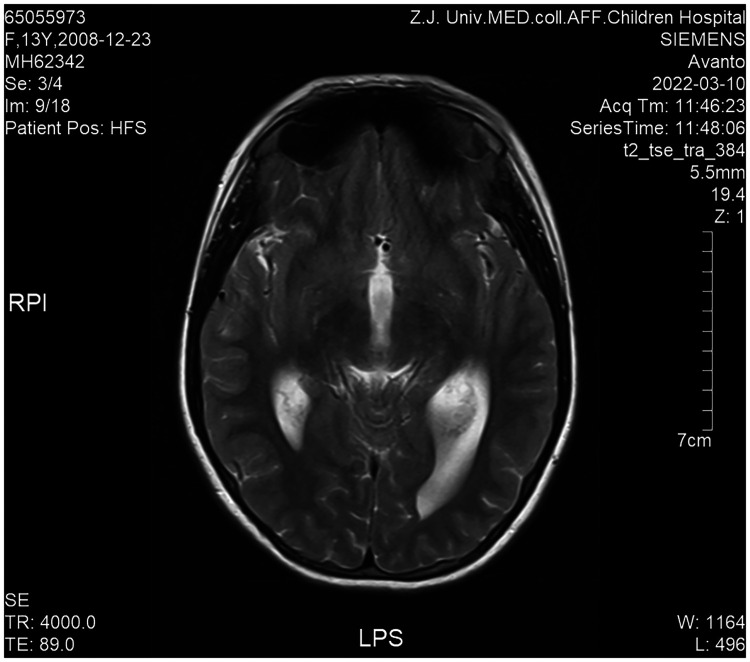
Brain MRI (sagittal position).

**Figure 4:**
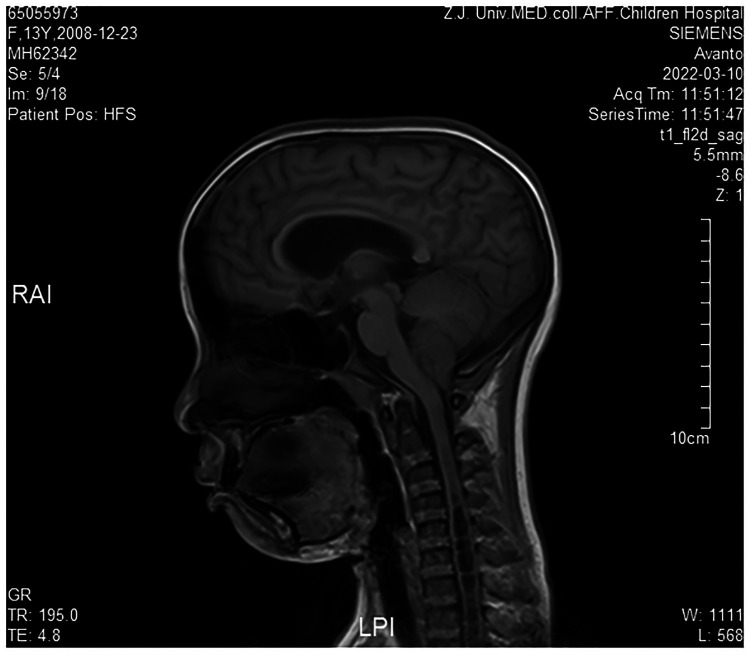
Brain MRI (transverse position).

Cranial magnetic resonance imaging (MRI) revealed Chiari **I** malformations, thinner corpus callosum, and enlarged supratentorial ventricles (Figure 2–4). Furthermore, MRIs of the cervical, thoracic, and lumbar cord all suggested extensive syringomyelia. Urinary ultrasound suggested enhanced medullary echogenicity in bilateral kidneys. The x-rays showed a bone age of 13 years old, similar to her chronological age. x-rays of both legs resulted as normal.

### 2.2. Whole exon sequencing (WES)

After the child’s guardians signed the informed consent form, peripheral blood (2 ml) was collected from the patient and her parents, and genomic DNA was extracted using the TIANamp Blood DNA Kit (Tiangen Biotech Co., China). DNA libraries were prepared according to Illumina’s protocol. Briefly, 3 μg of genomic DNA was fragmented by sonication (Covaris S2, USA). An “A” was then ligated to the 3′-end of the fragmented DNA. The sample was size-selected, aiming to obtain segments of 150∼250 bp for PCR amplification. Enriched libraries were then sequenced using an Illumina NextSeq 500 sequencer (Illumina, USA) for paired-end reads of 150 bp. Low-quality variations were filtered out using a quality score ≥ 20 and the BWA software package was used to align clean reads to the reference human genome (GRCh37). Single-nucleotide polymorphisms (SNPs) were identified using the SOAPsnp program (http://soap.genomics.org.cn/soapsnp.html), and insertions or deletions.

(InDels) were identified using GATK (http://www.broadinstitute.org/gsa/wiki/index.php/Home_Page). The identified SNPs and InDels were annotated using the Exome-assistant program (http://122.228.158.106/exomeassistant). SNPs and InDels with frequencies >0.01 in 1,000 Genome, ESP6500, ExAC_ALL, and ExAC_EAS were removed. Nonsynonymous variants were evaluated by Ployphen-2 (http://genetics.bwh.harvard.edu/pph2/), SIFT (http://sift.bii.a-star.edu.sg/), and MutationTaster (http://www.mutationtaster.org/) programs to predict their pathogenicity.

### 2.3. CNV-seq

We used the read-depth method to find duplications or deletions based on the density of reads on the chromosome. The basic principle is that duplicated regions have a higher density of reads than other regions, while deleted regions have a lower density of reads than other regions. After sequencing, the raw data were saved in a FASTQ format, then followed the bioinformatics analysis: First, Illumina sequencing adapters and low-quality reads (<80 bp) were filtered by Cutadapt (1.16) software (http://code.google.com/*p*/cutadapt/). After quality control, the clean reads were mapped to the UCSC hg19 human reference genome using BWA (0.7.12) software (http://bio-bwa.sourceforge.net/). Only uniquely mapped reads were selected. We used GATK (4.0.8.1) MarkDuplicates to remove duplicated reads; mapped reads were classified into adjustable sliding windows, which were 50 kb in length with 5 kb increments. The coverage of each window was calculated by the read amount and underwent two-step bias correction (GC correction and population-scale normalization); after correction, we used a binary segmentation algorithm to localize the segment breakpoints to identify the candidate CNV regions and determine the CNV genotype. We then used the U-test and Parallelism-test to estimate the genotype and significance of each segment. All suspected deletion or duplication regions were compared with the database, including OMIM, GeneReviews, Decipher, ClinVar, and DGV.

### 2.4. Mitochondrial gene sequencing (long-PCR + NGS)

Genomic DNA was extracted from peripheral blood and analyzed *via* next-generation sequencing (NGS). We performed mtDNA sequencing with GenCap® Mitochondrial Loop Gene Capture Probe V1.0 (MyGenostics, Beijing, China) using blood and samples. MtDNA sequencing analysis was performed with the latest Cambridge version of the mitochondrial genome (rCRS NC_012920) as the reference genome. Electrophoresis of mtDNA was also performed to exclude large-scale mtDNA deletions and rearrangements. The classification of variants followed the American College of Medical Genetics guidelines.

PCR amplification was carried out using the following primers (5′-3′): DNA2-F: AATAAGCTTT-CACTCATGCCAAG; DNA2-R: 142 AAGGATTCCTGATGCCATAGAAC. We used the Mutation Surveyor® software to compare the reference sequence with our sequencing data. The classification of variants followed the American College of Medical Genetics guidelines (ACMG).

### 2.5. Literature

Literature reports related to 11q13.3q13.4 regional microdeletions are from PUBMED and pathogenic/likely-pathogenic cases within the CNV region are from DECIPHER (Database of Chromosomal Imbalance and Phenotype in Humans using Ensembl Resources, https://www.deciphergenomics.org/), dbVar (https://www.ncbi.nlm.nih.gov/dbvar), Clingen (https://www.clinicalgenome.org/), and other databases. Disease-causing genes information is from OMIM (Online Mendelian Inheritance in Man, https://www.omim.org/), DECIPHER, and Clingen.

## 3. Results

CNV-seq based on WES analysis revealed a 2.10 Mb interstitial deletion spanning from 11q13.3 to 11q13.4, seq[hg19]del(11)(q13.3q13.4)chr11:g.69603687_71717599del, which was *de novo*. The mitochondrial gene shows no variations.

However, no clear pathogenic CNVs relevant to the phenotype were found in this region. The deleted interval contains 18 protein-coding genes (11 OMIM genes), 8 of which were closely related to diseases, including the SHANK2, DHCR7, NADSYN1, FADD, NUMA1, IL18BP, ANO1, and FGF3. Among them, SHANK2 is the only haplo-dosage-sensitive gene that has been proven with an HI (haploinsufficiency) score of 3. After searching the databases, some cases with a deletion region that overlapped with ours were found, most of which involved SHANK2 (Figure 2).

## 4. Discussion

Terminal 11q deletions have been widely reported to be associated with Jacobsen Syndrome (OMIM #147791). While reports of interstitial deletions between 11q13 and 11q23 are still scarce. In this study, we report a 2.11 Mb *de novo* deletion of chromosome 11q13.3–13.4 in a 13½-year-old girl and compare her phenotype and genotype with several previously described cases in order to point out specific candidate genes in our case within the deleted region.

Wischmeijer et al. first reported a *de novo* 3.4 Mb deletion of 11q13.2–13.4 (67,525,330 to 70,964,483 bp refer to Genome Assembly 2006, 67,768,754 to 71,286,835 bp refer to GRCh37/hg19) in an 8-year-old boy with global developmental delay, moderate to severe intellectual disability, severe speech delay, and some dysmorphic features, like deep-set eyes, ptosis, broad nasal bridge, and so on (Table 2) (4). It happens that there is a similar case, reported by Marcou et al., involving a 12-year-old girl with another *de novo* deletion of 11q13.2–13.4 (67,799,160 to 71,304,541 bp), presenting similar phenotypes including dysmorphic features, microcephaly, moderate to severe intellectual disability, and global developmental delay (Table 2) (5). The deleted regions in these two cases are quite similar and almost completely overlap, flanked by the same regions of segmental duplication (5). Among the genes involved, SHANK2 haploinsufficiency is considered to be relevant to these two patients’ phenotype of intellectual disability and severe language delay. Meanwhile, SHANK2 is the only haplo-dosage-sensitive gene with a score of 3 points, which means there is sufficient evidence for dosage pathogenicity. Our case provides additional evidence for the role of SHANK2 in intellectual disability, as described by previously reported cases.

**Table 2:** Clinical features of reported cases with deletions comprising chromosome 11q13.2–13.4.

References	Marcou et al. [2017]	Wischmeijer et al. [2011]	Kim et al. [2016]	Present case
Sex	Female	Female	Female	Female
Age when reported	12	$8\frac{1}{2}$	1	$13\frac{1}{2}$
Deleted region (range)	11q13.2–13.4	11q13.2–13.4	11q13.2–13.3	11q13.3–13.4
Deletion size, Mb	3.4	3.4	2.75	2.11
Development	Global developmental delay	Developmental delay, moderate-severe intellectual disability	Moderate developmental delay, severe mental development	Global developmental delay, severe intellectual disability
Speech	Severe language delay	Severe speech delay		Speech delay
Microcephaly	+	–	+	–
Forehead	Prominent forehead	Prominent forehead		
Face features	Plagiocephaly with mild facial asymmetry, mild malar hypoplasia, mild retrognathia	Short philtrum		Long, deep philtrum
Eyes	Bilateral epicanthal folds with deep-set eyes, long palpebral fissures	Ptosis, strabismus, astigmatism, downward slanting palpebral fissures, deep-set eyes	Ptosis of the left eye	Ocular hypertelorism, thick eyebrows
Nose	An upturned nose, a broad nasal tip, depressed nasal bridge	Broad nasal bridge, tubular somewhat beaked nose with round overhanging tip and hypoplastic nares		Broad nasal tip with anteverted nares
Ears	Cup-shaped anteriorly protruding ears, low-set ears, petite, long with over-folding of the superior helix.	Low-set posteriorly rotated ears with overfolded helices and a preauricular tag at the left side	Right auricular deformity, right microtia (small ear) with shortened upper parts of the auricles	
Mouth	Small mouth, slight down-turned corners	Small, thin upper lip	High-arched palate	Thick lower lip
Teeth	Wide-spaced teeth	Small and central incisors widely spaced	Dentition was delayed	
Hand	Long slender fingers, hypermobility in the small joints	Hands were narrow with long slender fingers, clinodactyly of both fifth fingers	Simian line on the right hand	
Foot	Long toes, thin slender feet, mild pes planus	Metatarsus adductus, partial cutaneous syndactyly of toes II-III		
Others	Hypotonia, mild pectus excavatum	The right nipple appeared slightly enlarged and elevated, and hypertrichosis was apparent on her back and lower limbs.		Clitoral hypertrophy, proximal tubular acidosis, hypophosphatemia rickets

SHANK2 is located at 11q13.3–q13.4, genomic coordinates (GRCh37): chr11:70,313,961–70,935,842, composed of 25 exons. The protein it encodes is a member of the postsynaptic scaffolding protein family (currently compromising three members, SHANK1, SHANK2, and SHANK3). They are all localized in the postsynaptic regions of excitatory synapses in the brain, essential for the growth and function of synapses (6). Mutations in the SHANK2 gene have been well-described in individuals with autism spectrum disorder (ASD) and intellectual disability (ID). Through the genome-wide microarray scanning of 184 unrelated individuals with ID and 396 individuals with ASD, respectively, Berkel et al. discovered one patient in each cohort with a copy number variant: 120-kb and 69-kb deletion in the SHANK2 gene, both patients had ASD of comparable severity and mild to moderate intellectual disability (7). On this basis, to explore the link between the *de novo* SHANK2 mutation and ASD in humans, Won et al. generated transgenic mice carrying the same mutation as the ASD-associated microdeletion in the human SHANK2 gene (exon 6 and 7 deletion and a frameshift), resulting in mice exhibiting ASD-like behaviors, including reduced social interaction and reduced social communication through ultrasonic vocalizations and repetitive jumping, suggesting that SHANK2 may significantly affect neurocognitive function (8).

In addition to the previously reported cases, another case with a deletion of a 2.75 Mb segment in the region spanning from 67,524,823 to 70,272,728 in 11q13.2–q13.3 was reported by Kim et al. (9). In this case, a 1-year-old girl presented with delayed dentition, moderate developmental delay, and some craniofacial dysmorphic features (Table 2). They considered that FGF3 is a possible candidate gene. In general, mutations in FGF3 are associated with an autosomal recessive disease: deafness, congenital with inner ear agenesis, microtia, and microdontia (OMIM:610706). However, Gregory-Evans et al. have proposed that FGF3 haploinsufficiency could contribute to otodental syndrome (OMIM:166750), which is a rare but severe autosomal dominant craniofacial anomaly, characterized by grossly enlarged canine and molar teeth (globodontia) along with a high-frequency sensorineural hearing deficit (10). A case listed in DECIPHER (Patient 2389) carried a partially overlapping deletion spanning from 68,548,275 to 70,783,506 bp refer to GRCh38/hg18, 68,315,743–70,629,611 bp refer to GRCh37/hg19 and presented congenital deafness as well. In our case, we didn’t observe any dental abnormalities or detect hearing loss. However, since the onset of hearing loss may vary from early childhood to middle age, we think a long-term follow-up is required.

Aside from SHANK2, FGF3, the deleted region of our patient contains other genes that are known to be disease-causing, but some of them are responsible for autosomal recessive disorders: FADD (Immunodeficiency 90 with encephalopathy, functional hyposplenia, and hepatic dysfunction, OMIM #613759), DHCR7 (Smith-Lemli-Opitz syndrome, OMIM #270400), NADSYN1 (Vertebral, cardiac, renal, and limb defects syndrome 3, OMIM #618845), and IL18BP (Hepatitis, fulminant viral, susceptibility to, OMIM #618549). Since these syndromes are autosomal recessively inherited and our patient showed no typical clinical signs, we excluded the effects of these genes.

The renal manifestations in our patient are relatively unique and have not been reported in cases within the surrounding regions, which can be clinically diagnosed as Fanconi renotubular syndrome (FRTS). Fanconi renotubular syndrome, also referred to as Fanconi-Debré-de Toni syndrome, since Debré, de Toni, and Fanconi took the lead in describing a series of cases of rickets, glycosuria, and albuminuria (11). FRTS is a renal tubular reabsorption disorder caused by a genetic or acquired cause, with clinical manifestations including proximal tubular acidosis, glycosuria, aminoaciduria, hyperphosphaturia (hypophosphatemia), carbonaturia, hypokalemia, and hyponatremia (12). Here, we list several genetic etiologies of FRTS (Table 3), comprising systemic disorders and five forms of isolated FRTS (FRTS type 1–5). The pathogenic mechanisms include the excessive accumulation of a toxic metabolite due to mutations in genes encoding carrier proteins or enzymes in metabolic processes, disruption of receptor-mediated cellular endocytosis, and impaired energy metabolism. However, according to the results of gene detection, our patients do not have any variants of the genes we listed. While acquired FRTS is often secondary to diseases like multiple myeloma (25), Sjogren’s syndrome (26), etc., or exposure to certain toxins or drugs (the most frequently implicated drugs, including anti-cancer agents, anti-virals, anti-epileptic drugs, and aminoglycoside antibiotics such as cisplatin, ifosfamide, tenofovir, adefovir, sodium valproate, etc.) (27, 28). However, our patient does not have a history of taking these drugs, nor does she have a history of diseases that could contribute to FRTS.

**Table 3:** Genetic forms of fanconi syndrome.

Inherited systemic disorders		
Cystinosis (OMIM#219800)	CTNS (17p13.2)	CTNS gene encodes a lysosomal cystine/proton symporter termed cystinosin. Cystine accumulates and crystallizes in the lysosomal lumen without cystinosin (13).
Hypertyrosinaemia type1 (OMIM#276700)	FAH (15q25.1)	FAH encodes, the last enzyme in tyrosine degradation. The deficiency of fumarylacetoacetase results in the accumulation of the toxic agents fumaryl- and maleylacetoacetate (14).
Fanconi–Bickel syndrome (OMIM #227810)	SLC2A2 (3q26.2)	SLC2A2, which is also called GLUT2, encodes facilitative glucose transporter 2. Mutations in the SLC2A2 gene impair glucose, metabolism leading to glycogen accumulation (15).
Fructose intolerance (OMIM #229600)	ALDOB (9q31.1)	ALDOB encodes aldolase B (fructose-bisphosphate aldolase), an enzyme that brings about the assimilation of fructose with cleavage of fructose-1 phosphate to trioses. Affected people suffer from episodic hypoglycemia, liver disease, renal tubular acidosis, and growth retardation when exposed to fructose (16).
Wilson’s disease (OMIM #277900)	ATP7B (13q14.3)	ATP7B encodes a polypeptide that acts as a plasma membrane copper-transport protein. A defect of copper transport by the hepatic lysosomes leads to excess deposition of copper in the organs (17).
Lowe syndrome (OMIM# 309000)	OCRL (Xq26.1)	Lowe syndrome has been defined as the triad of congenital cataracts, intellectual disability, and renal Fanconi syndrome. OCRL1 encodes a phosphatidylinositol bisphosphate 5-phosphatase (PIP2P), which has been shown to regulate vesicular transport. Mutation in OCRL affects endosomal trafficking (18).
Dent disease I/II (OMIM#)	CLCN5/OCRL (Xp11.23/Xq26.1)	CLCN5 encodes the CLC-5 chloride channel, a lysosomal voltage-gated chloride transporter that acidifies the endosomes through an ATPase pump to allow the exchange of a chloride for a proton. Inactivation of the CLC-5 leads to generalized dysfunction of the proximal tubule (15). In addition to Lowe syndrome, the OCRL mutation also causes Dent syndrome, which has similar renal manifestations but no ocular or cerebral involvement in Dent syndrome. These two diseases differ in the distribution of mutations in the OCRL gene (18).
Isolated Fanconi syndrome		
FRTS1 OMIM #134600	GATM (15q21.1)	GTAM encodes glycine amidinotransferase, which is a renal proximal tubular enzyme in the creatine biosynthetic pathway. Mutations in the gene create an additional protein-protein interaction surface, where the protein gathers into large aggregates. These aggregates were associated with increased reactive oxygen species (ROS) production, inflammatory signals, cell death, and renal fibrosis (19).
FRTS2 OMIM #613388	SLC34A1 (5q35.3)	SLC34A1 encodes sodium phosphate co-transporter NaPi-IIa, which is key to maintaining whole-body phosphate homeostasis. Mutations in the gene cause phosphate wasting along with insufficient ATP production (20, 21).
FRTS3 OMIM #615605	EHHADH (3q27.2)	EHHADH encodes the bifunctional peroxisomal enzyme enoyl-CoA hydratase–L-3-hydroxyacyl-CoA dehydrogenase, also called L-bifunctional enzyme or L-PBE, which involves peroxisomal fatty acid beta-oxidation. The mistargeting of EHHADH functionally disrupts some multimers of the mitochondrial trifunctional enzyme, which has a high degree of homology to EHHADH and also plays a role in mitochondrial fatty acid oxidation and, thus, affects mitochondrial energy metabolism and impairs reabsorption in the renal proximal tubules (22).
FRTS4 OMIM #616026	HNF4A (20q13.12)	HNF4A encodes hepatocyte nuclear factor 4, which is well-known to be associated with maturity-onset diabetes of youth (MODY) type 1 (OMIM# 616026). A specific heterozygous R76W mutation affects mitochondria by decreasing the transcription of genes for mitochondrial structure and function, along with a reduction in beta-oxidation, leading to lipid droplet accumulation (23).
FRTS5 OMIM #134600	NDUFAF6 (8q22.1)	Mutation in NADH:ubiquinone oxidoreductase complex assembly factor 6 (NDUFAF6), which leads to aberrant splicing of NDUFAF6 mRNA and loss of the mitochondria located NDUFAF6 isoform, resulting in NADH: ubiquinone oxidoreductase complex (mitochondrial respiratory chain complex I) deficiency. This type only occurs in Acadians presented with proximal renotubular dysfunction from birth, followed by progressive kidney disease and pulmonary interstitial fibrosis (24).

We hypothesized that ANO1 may be the candidate gene for the presentation of FRTS in our case. ANO1 encodes calcium-activated chloride channel (CACC), which is a transmembrane protein. Faria et al. have identified that ANO1 was predominantly expressed in the apical membrane of proximal tubular epithelial (PTE) cells (29). As we know, the proximal tubule is the main place for reabsorbing various filtered electrolytes as well as low-molecular-weight proteins (relative molecular weight <50,000), amino acids, glucose, and urate (30). Mice with selective tubular knockout of Ano1 (Ano1^lox/lox^/Ksp-Cre) exhibited proteinuria (mainly LMW proteins); as we know, proteinuria can be caused by defects in the glomerular filter or by impaired reabsorption of filtered proteins in the proximal tubule. Researchers have found an accumulation of endosomal storage vesicles at the apical pole of PTE cells in ANO1-null kidneys, which was not observed in WT mice, whereas structural and functional changes in glomerular filters and podocytes were not observed. The results were also confirmed in mice with knockout of Ano1 in podocytes (Ano1^lox/lox^/Nphs2-Cre). The authors suggested the deficit of ANO1 may affect proton secretion and reduced endosomal acidification, leading to impairment of reabsorption (29). Recently, Schenk et al. have observed a reduced nephron count and histological signs of tubular damage in TMEM16A (ANO1) knockout animals and hypothesized that ANO1 may play a significant role in nephrogenesis at first, then secondly cause damage to proximal tubular cells and subsequently, loss of cilia and defective endocytic uptake of low-molecular weight proteins (31). The published literature only describes the relationship between ANO1 and proteinuria due to tubular damage, but the link between ANO1 and the pathogenesis of FRTS is not clear at present. However, these findings were a boost to our further investigation, especially focusing on urine pH, urinary glucose, and urine electrolyte concentrations of ANO1 knockout animals. Of note, the potential function of the remaining genes in the deleted region is currently unclear. Therefore, the renal manifestation may be the result of the combined effect of other haploinsufficiency genes within the deleted region.

Since phosphorus is an essential component for the mineralization of bone, hypophosphatemia rickets is thought to be secondary to increased renal phosphate excretion when hereditary factors (such as X-linked hypophosphatemia rickets) have been ruled out (32). Rothenbuhler et al. have reported that 61% of children with X-linked hypophosphatemic rickets (XLHR) show either craniosynostosis and/or a Chiari I malformation (33). A similar finding is also seen in an article by Caldemeyer and colleagues, which reported that Chiari I malformation occurred in 7 of 16 patients with hereditary hypophosphatemic rickets (HHPR) (34). These studies indicate that calcium and phosphate metabolism dysregulation may be a significant factor in the development of Chiari I malformation, which may explain that Chiari malformation is secondary to hypophosphatemic rickets.

Array-based Comparative Genomic Hybridization (aCGH), Fluorescence *in situ* Hybridization (FISH), and New Generation Sequencing (NGS) are the main methods used for CNV analysis. In this case, we first detected CNVs from WES data using read-depth-based detection, and the result was then confirmed by NGS CNV-seq. At present, WES is often the first-line genetic study used in clinical practice since single gene disorders are the most common. Our case indicated that WES-based CNV analysis could provide an additional method of CNV detection, further improving the diagnostic rate in rare genetic diseases.

In conclusion, we identified a *de novo* 2.14 Mb deletion from 11q13.3 to 11q13.4 in a patient with global developmental delay, intellectual disability, and Fanconi renotubular syndrome. It is the first case of chromosome 11q13.3 to 11q13.4 microdeletion with some phenotypic overlap with previously reported cases of deleted range from 11q13.2 to 11q13.4. The renal manifestation of our patient expands the clinical spectrum resulting from a microdeletion of this region. Nevertheless, additional descriptions of more patients with microdeletion 11q13.3 to 11q13.4, and continued investigations are needed to further understand the function of deleted intervals and explore a genotype-phenotype correlation.

## Data Availability

The datasets for this article are not publicly available due to concerns regarding participant/patient anonymity. Requests to access the datasets should be directed to the corresponding author.
